# Combined In Situ EQCM‐Raman Study of Zn Storage Mechanism in Polyaniline for Zinc‐Ion Battery

**DOI:** 10.1002/smtd.202501273

**Published:** 2025-10-01

**Authors:** Emine Kapancik Ulker, Pranay Hirani, Shaoliang Guan, Abhishek Lahiri

**Affiliations:** ^1^ Department of Chemical Engineering Brunel University London Uxbridge UB8 3PH UK; ^2^ Department of Chemistry Faculty of Science and Arts Recep Tayyip Erdogan University Rize 53100 Turkey; ^3^ Maxwell Centre Cavendish Laboratory J J Thomson Avenue Cambridge CB3 0HE U.K.

**Keywords:** doping, electropolymerization, in situ eqcm‐raman spectroscopy, polyaniline, Zn‐ion batteries

## Abstract

Zinc‐ion batteries (ZIBs) have attracted increasing attention as safe, cost‐effective, and environmentally friendly alternatives to lithium‐ion batteries for large‐scale energy storage. Among various cathode materials for Zn batteries, polyaniline (PANI) is a potential material that presents benefits such as high conductivity and pseudocapacitive behavior. However, it often suffers from limited cycling stability and structural degradation during repeated charge–discharge processes. Here, in situ electrochemical quartz crystal microbalance (EQCM)‐Raman technique is combined to understand the Zn storage behavior in PANI wherein limited Zn insertion is observed along with detachment of the polymer from the substrate. Through anion doping of PANI, the structural stability is enhanced, and the overall Zn cycling capability is improved. Ex situ X‐ray photoelectron spectroscopy (XPS) studies further reveal that doping of PANI significantly reduces the oxidation of PANI, which leads to an improved battery performance. The doped‐PANI shows a high specific capacity of 310 and 235 mAh g^−1^ at 0.25 and 1 A g^−1^, respectively, and retains 85% of its initial capacity after 300 cycles at 1 A g^−1^. These results reveal that it is important to understand the storage mechanism to develop useful strategies to improve ZIBs performance.

## Graphical abstract

A new combined EQCM‐Raman analysis method of Zn storage mechanism in polyaniline is presented, from which changes in the polyaniline structure due to Zn insertion/extraction and doping/dedoping are revealed. By anionic doping of polyaniline, the capacity and stability of Zn‐ion batteries showed substantial improvement.

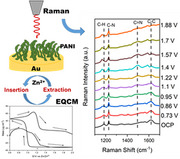

## 1 Introduction

Among next‐generation energy storage systems, Zn‐ion batteries are gaining considerable attention as a cost‐effective and environmentally benign alternative to conventional lithium‐ion and other metal‐ion batteries, particularly for grid‐scale and portable energy storage applications.^[^ ^1^, ^2^ ^]^ ZIBs have several inherent advantages, including low material cost, relatively high theoretical capacity (820 mAh g^−1^), the intrinsic safety of Zn metal, compatibility with aqueous electrolytes, and recyclability.^[^ ^3^ ^]^ However, the commercial viability of ZIBs remains limited due to several critical challenges, such as inefficient and unstable zinc storage at the cathode, the formation of Zn dendrites during charge–discharge cycles, low coulombic efficiency, and the dissolution of cathode materials into the electrolyte. To address these challenges, current research has focused on the development of novel cathode materials such as metal oxides, conducting polymers, and carbon‐based materials, which have been studied in aqueous electrolytes in ZIBs.^[^ ^4^, ^5^, ^6^ ^]^

Conductive polymers, particularly polyaniline, have several advantages as cathode materials, including good electrical conductivity due to their extended π‐electron conjugation, structural flexibility, environmental stability, and ease of synthesis.^[^ ^7^ ^]^ PANI is a well‐known conducting polymer with notable electrochemical properties, attributed to its ability to reversibly switch between different oxidation states: leucoemeraldine (fully reduced), emeraldine (partially oxidized), and pernigraniline (fully oxidized).^[^ ^8^, ^9^ ^]^ These redox transitions involve changes in the polymer backbone along with ion doping/dedoping to maintain charge balance. In aqueous ZIBs, most studies focus only on the leucoemeraldine‐to‐emeraldine transition, as further oxidation to pernigraniline requires higher potentials that exceed the electrochemical window of water. Moreover, pernigraniline is unstable in aqueous environments and tends to degrade via hydrolysis, leading to chain splitting and loss of active material, which causes capacity fading. To prevent this, the upper voltage limit is often restricted, but this also limits the utilization of PANI's full capacity.^[^ ^10^, ^11^ ^]^ Enabling the complete redox transition across all three states could significantly improve the energy storage performance of PANI‐based electrodes.^[^ ^12^ ^]^ Furthermore, the electrochemical performance of PANI is hindered by its poor cycle stability and the deficiency of active sites due to its spontaneous deprotonation processes and structural irregularities within the polymer matrix.^[^ ^13^ ^]^ Generally, some strategies such as adjusting morphology,^[^ ^14^, ^15^, ^16^ ^]^ introducing conductive carbon materials,^[^ ^17^ ^]^ and heterostructure construction^[^ ^18^ ^]^ are employed to improve the performance of PANI. Doping of PANI with various ions, including Ni^2+^,^[^ ^19^ ^]^ Mg^2+^,^[^ ^18^ ^]^ Co^2+^,^[^ ^20^ ^]^ H^+^,^[^ ^13^ ^]^ Zn^2+^,^[^ ^21^, ^22^ ^]^ has emerged as a promising way to enhance its electrochemical and structural properties, particularly for applications in energy storage. Doping directly modifies the electronic structure of the PANI backbone, facilitating improved charge carrier mobility and redox activity. Studies have shown that M^2+^‐doped PANI films exhibit higher specific capacitance and better cycling stability than their undoped counterparts, likely due to increased ionic conductivity and more efficient charge transfer pathways.^[^ ^18^, ^19^, ^20^, ^21^, ^22^ ^]^ Introducing M^2^⁺ can also lead to morphological changes in the polymer matrix, such as increased porosity or nanostructure, which further enhances ion diffusion during electrochemical processes.^[^ ^19^ ^]^ As a result, M^2+^‐doped PANI presents a viable approach to tuning the performance of conductive polymers without relying on inorganic oxide additives. In addition, the incorporation of the anionic dopants, such as SO_4_^2−^, SO_3_^2−^, and Cl^−^, during anodic polymerization is also critical for stabilizing the positively charged backbone of PANI and maintaining its conductivity.^[^ ^7^, ^23^, ^24^, ^25^, ^26^ ^]^ These anions compensate the positive charges formed on the nitrogen atoms during oxidation, enabling the formation of delocalized polaronic and bipolaronic species that are responsible for electronic conductivity. The size and the type of the anionic dopant can also influence the morphology and the stability of the polymer.^[^ ^24^, ^25^ ^]^ Thus, both cationic and anionic doping provide complementary pathways tuning the electrochemical behavior of PANI, and their synergistic effects are particularly advantageous for optimizing its performance in ZIBs.

In this study, PANI and anion‐doped PANI films were synthesized by a facile cyclic voltammetry method in an acidic solution and investigated as cathode materials for aqueous ZIBs to address the limitations of pristine PANI in terms of cycling stability and capacity retention. A combined in situ electrochemical quartz crystal microbalance (EQCM)‐Raman setup was employed to gain real‐time insight into the Zn storage mechanism, which was further supported by ex situ X‐ray photoelectron spectroscopy (XPS) studies. Unlike studies that utilized either EQCM or Raman individually or relied on ex situ approaches, our integrated method simultaneously tracks mass changes and molecular structure evolution on the same electrode during electrochemical cycling. This enables direct correlation of Zn^2+^/triflate (TfO^−^) ion dynamics with redox‐induced structural transitions within the PANI matrix. Structural changes in PANI, along with a distinct Zn storage mechanism, were found to contribute to the improved electrochemical performance. To the best of our knowledge, this is the first report of integrating EQCM and Raman techniques in situ for investigating Zn ion interactions in conducting polymer systems, highlighting the potential of this methodology for advanced electrochemical material studies.

## 2 Results and Discussion

### 2.1 EQCM Studies on the Electrodeposition of PANI

**Figure 1:**
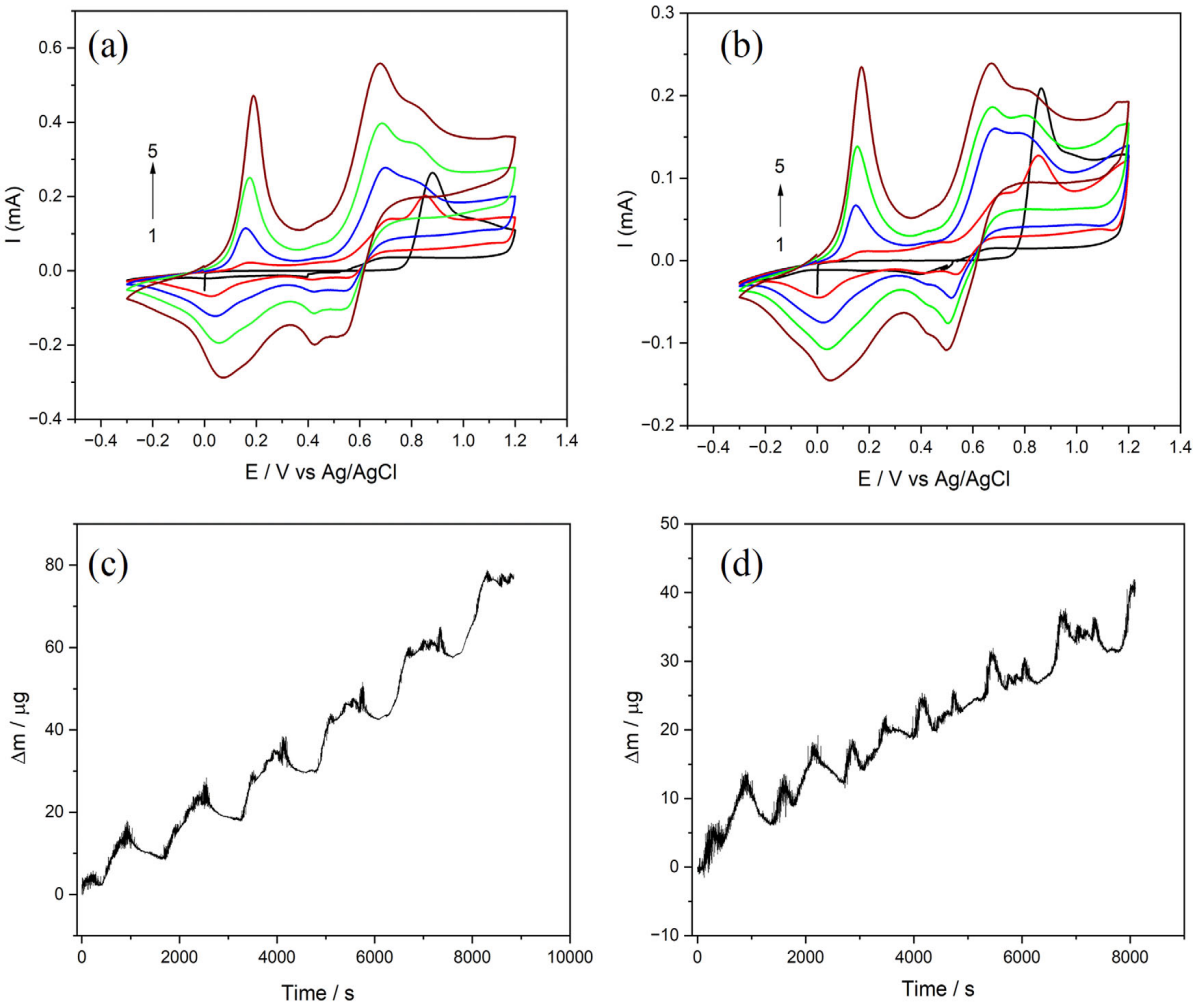
I vs E; and Δm vs time from EQCM data for the deposition of PANI (a,c) and PANI/TfO_25_ (b,d) film from −0.5 to 1.2 V at 1 mV s^−1^.

The deposition of PANI and doped‐PANI films was studied using EQCM in the potential range from −0.5 to 1.2 V vs Ag/AgCl. **Figure** **1**a,b compares the CV of undoped and doped‐PANI on Au quartz crystal. It is evident from the CV that during the first cycle, the aniline monomer undergoes oxidation at ≈0.90 V. During subsequent polymerization of PANI films, three redox peaks are observed: the first peak at ≈0.15 V corresponds to the formation of cation‐radicals; the second, a smaller peak at ≈0.40 V, is attributed to the generation of intermediates and by‐products; and the final peak near 0.70 V is associated with the growth of polymer chains.^[^ ^27^ ^]^ In both solutions, the redox current increases with successive cycles in aqueous media. However, the current observed in the Zn(TfO)_2_ containing solution is nearly half of that in the Zn‐free electrolyte, suggesting that Zn^2+^ ions interfere with the electron transfer kinetics of aniline oxidation and/or hinder the subsequent growth of the polymer chains. In the first scan, the mass of the PANI film increased upon reaching the monomer's oxidation potential at 0.9 V (Figure S1, Supporting Information). In subsequent scans, although the oxidation potential shifted to 0.7 V in the CV (Figure 1a), indicating the growth of the polymer chain, a nearly constant mass increase was observed in each cycle (Figure S1, Supporting Information). In the Zn‐containing solution, a similar behavior is observed during film growth. However, a distinct mass increase is also detected during the cathodic scan, particularly after the second cycle. This is thought to be associated with the incorporation of TfO^−^ into the polymer structure (Figure S2, Supporting Information). Figure 1c,d display the time‐dependent mass increase of the PANI and doped‐PANI films, respectively. The presence of a second, sharp but small peak indicates the possible insertion of TfO^−^ into the polymer structure during the growth of the PANI film (Figure 1d). Although both films exhibit a regular growth over time, the mass increase observed in the TfO‐containing film is lower compared to that of the PANI film. In both PANI and doped‐PANI films, the change in mass observed at each cycle in EQCM measurements can be primarily attributed to the doping/dedoping processes, wherein previously incorporated anions are expelled from the polymer matrix as the film undergoes reduction. This is a characteristic behavior of conducting polymers, where electrochemical reduction leads to the release of charge‐balancing dopant ions and associated solvent molecules.^[^ ^28^ ^]^ In the case of the doped‐PANI the mass loss appears more pronounced, which may indicate the additional release or rearrangement of ions initially incorporated into the polymer structure.

### 2.2 Structural and Morphological Characterizations

**Figure 2:**
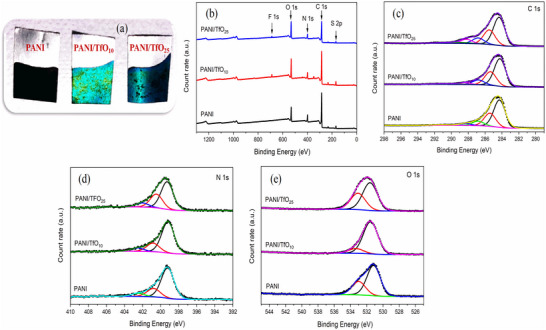
a) Picture of PANI and doped‐PANI electrodes, b) Comparison of XPS survey scans of the PANI and doped‐PANI films in the binding energy range of 0–1300 eV (the main peaks for the elements detected in the films are identified), detailed c) C 1s, d) N 1s and e) O 1s spectra of the PANI and doped‐PANI films.

The morphological, structural, and chemical compositional properties of the PANI and doped‐PANI electrodes were investigated by SEM and XPS measurements. Electrodeposited PANI on graphite paper shows a black coloured deposit (**Figure** **2**a). At low concentrations of Zn(TfO)_2_ in the electrolyte, the electropolymerized PANI film exhibited a green–black coloured deposit, which is typical for the emeraldine oxidation state. However, as the Zn(TfO)_2_ concentration increased, a gradual color transition from green to blue was observed (Figure 2a), indicating possible changes in the oxidation level and chain conformation of the polymer. SEM images of PANI and doped‐PANI, presented in Figure S3 (Supporting Information), clearly illustrate the influence of Zn(TfO)_2_ on the morphology and porosity of the film. While PANI synthesized in H_2_SO_4_ exhibits a globular morphology, the introduction of the Zn(TfO)_2_ into the electrolyte significantly alters its structure, leading to a more heterogeneous surface.

XPS analysis was used to investigate the changes in the polymer composition. Figure 2b compares the XPS survey spectra of the PANI, and doped‐PANI (doped PANI solution containing 10 mM Zn(TfO)_2_ represented as PANI/TfO_10_ and PANI solution containing 25 mM Zn(TfO)_2_ represented as PANI/TfO_25_) films. The XPS spectra of the films reveal the presence of sulfur (S 2p, 167.6 eV), carbon (C 1s, 285.4 eV), nitrogen (N 1s, 400 eV), and oxygen (O 1s, 532.5 eV). The S comes from the SO_4_^2−^ and (TfO)^−^ anions used in the electrolyte to deposit the polymers. In addition, the F 1s peak at 689.9 eV was only observed in the doped‐PANI films synthesized with Zn(TfO)_2_, indicating the presence of fluorine originating from the (TfO)^−^ anion. Figure 2c–e shows the detailed spectra of C 1s, N 1s, and O 1s, reflecting the changes in the polymer films after doping. In all films, C 1s spectra typically exhibit peaks at 284.3 eV corresponding to the binding energy of the aromatic C─C and C═C, with additional components ≈285.4, 286.8, and 288.3 eV attributed to the C─N, C═O (C─N^+^), and COO binding energy (Figure 2c).^[^ ^13^, ^29^ ^]^ In addition, the C–F peak observed at 288.3 eV in the PANI/TfO_25_ sample (Figure S4, Supporting Information) exhibits a higher intensity compared to that of PANI/TfO_10_, indicating a greater incorporation of fluoride into the PANI matrix as a result of the higher concentration of Zn(TfO)_2_. In the N 1s XPS spectrum of the samples, three main peaks were observed at 399.2, 400.7, and 402.5 eV corresponding to quinoid imine (─N═), benzenoid amine (─NH─), and the positively charged polaron species (─NH^+^═), respectively (Figure 2d).^[^ ^26^ ^]^ In the O 1s XPS spectra, two distinct peaks were observed at 531.5 and 533.0 eV, corresponding to different oxygen‐containing species on the PANI surface (Figure 2e). The peak at 531.5 eV is typically attributed to carbonyl groups (C═O), while the 533.0 eV peak is associated with adsorbed oxygen.^[^ ^30^ ^]^

Based on the structural and morphological characterizations of PANI deposits, it is evident that Zn(TfO)_2_ significantly alters the properties of PANI films. The results suggest that Zn(TfO)_2_ induces coordination‐based doping, leading to modification in the electronic structure of PANI, and offering opportunities for tuning its properties in energy applications.

### 2.3 In Situ EQCM‐Raman Analysis

**Figure 3:**
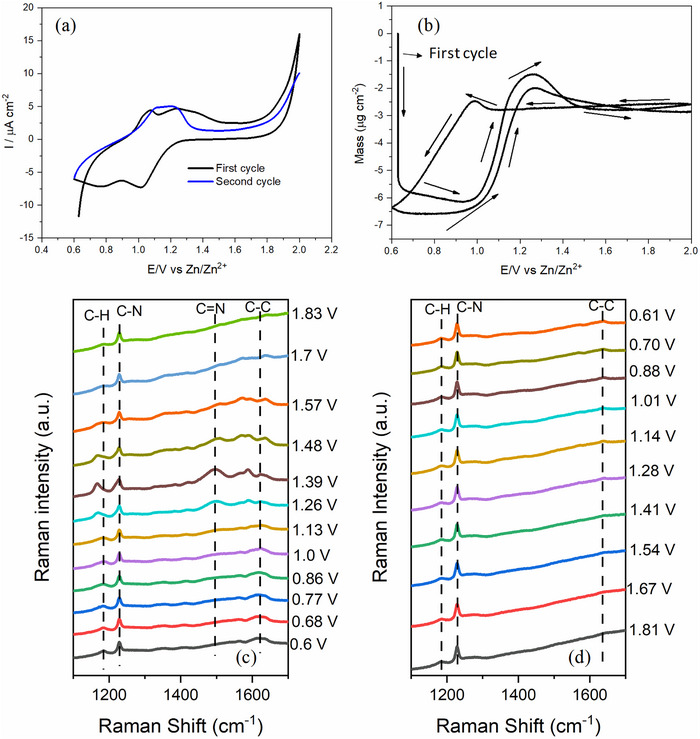
a) Cyclic voltammogram and b) corresponding mass change of Zn interaction with PANI during cyclic voltammetry in the potential range of 0.6–2.0 V in 2 m Zn(TfO)_2_ electrolyte; c,d) Corresponding in situ Raman spectra of PANI during CV.

To understand the Zn storage mechanism in PANI, in situ EQCM‐Raman measurements were carried out during charge and discharge processes. **Figure** **3** presents the cyclic voltammogram, EQCM, and Raman measurements performed simultaneously on electrodeposited PANI on an Au quartz crystal. The CV profile of Zn interaction with PANI typically exhibits two redox pairs at ≈0.9 and 1.1 V, which are indicative of its redox transitions among different oxidation states. These peaks represent the reversible electrochemical conversions between leucoemeraldine, emeraldine, and pernigraniline forms of PANI along with Zn interaction (Figure 3a). The current increase observed at ≈1.8 V can be attributed to the oxygen evolution reaction (OER), which may be due to the catalytic activity of the Au electrode beneath the thin PANI layer. The corresponding mass changes recorded during CV are presented as a function of potential in Figure 3b. A slight mass decrease observed at the beginning of the anodic scan can be attributed to strain‐induced removal of the loosely bound polymer from the electrode surface.^[^ ^31^ ^]^ Upon oxidation of PANI at 1.0 V, a significant mass increase was observed up to 1.3 V, attributed to the insertion of the TfO^−^ anion into the oxidized polymer matrix to maintain charge neutrality. Beyond 1.3 V, the mass plateaued, suggesting saturation of anion uptake. During the reverse scan, a minor mass increase was observed at 1.0 V, which may be attributed to the incorporation of Zn^2+^ ions into the polymer matrix. This suggests that, in addition to the triflate anion during anodic scan, Zn^2+^ cation may also participate in the charge compensation or interact with nitrogen sites on the polymer backbone. Subsequently, during the cathodic scan ≈0.9 V, anion expulsion leads to a corresponding decrease in the mass. Figure 3c,d shows the in situ Raman spectra at different electrode potentials during anodic and cathodic scans, respectively. From Figure 3c, it can be seen that Raman spectrum at initial potential contains characteristic peaks of PANI, which are located at 1184 cm^−1^ (C─H bending of the benzenoid ring), 1227 cm^−1^ (quinoid‐ring deformation vibration), 1347 cm^−1^ (C─N^+^ stretching in polaronic form), 1561 cm^−1^ (C─C stretching in benzenoid ring) and 1620 cm^−1^ (C─C stretching vibrations of the benzenoid ring), suggesting the predominance of the emeraldine salt form.^[^ ^32^, ^33^ ^]^ Initially, there is no peak at 1493 cm^−1^, which corresponds to C═N stretching vibrations in quinonoid units. Upon increasing the potential from 1.26 to 1.57 V, a distinct band emerges at 1496 cm^−1^, signifying the PANI oxidation to pernigraniline form. The disappearance of C═N band in PANI at high potentials may be attributed to the degradation of the polymer backbone due to overoxidation (Figure 3c)^[^ ^34^ ^]^ During anodic scan, the C–N stretching band at 1227 cm^−1^ displays broadening, while the C–H bending vibration at 1184 cm^−1^ shifts to the lower wavenumbers as the potentials increase. Meanwhile, a new peak emerges at 1586 cm^−1^ near the C═C stretching band at 1561 cm^−1^. The gradual appearance and growth of this quinoid‐associated band alongside the benzenoid peak indicate an oxidative transformation within the polymer backbone. In the reverse scan, no significant changes were observed in the Raman spectrum, indicating that the structural transformation induced during the anodic scan is largely retained (Figure 3d). This suggests that the quinoid structures formed at higher potentials remain stable during the reverse process, and the polymer does not undergo a complete transition back to its reduced benzenoid form under the applied conditions.

**Figure 4:**
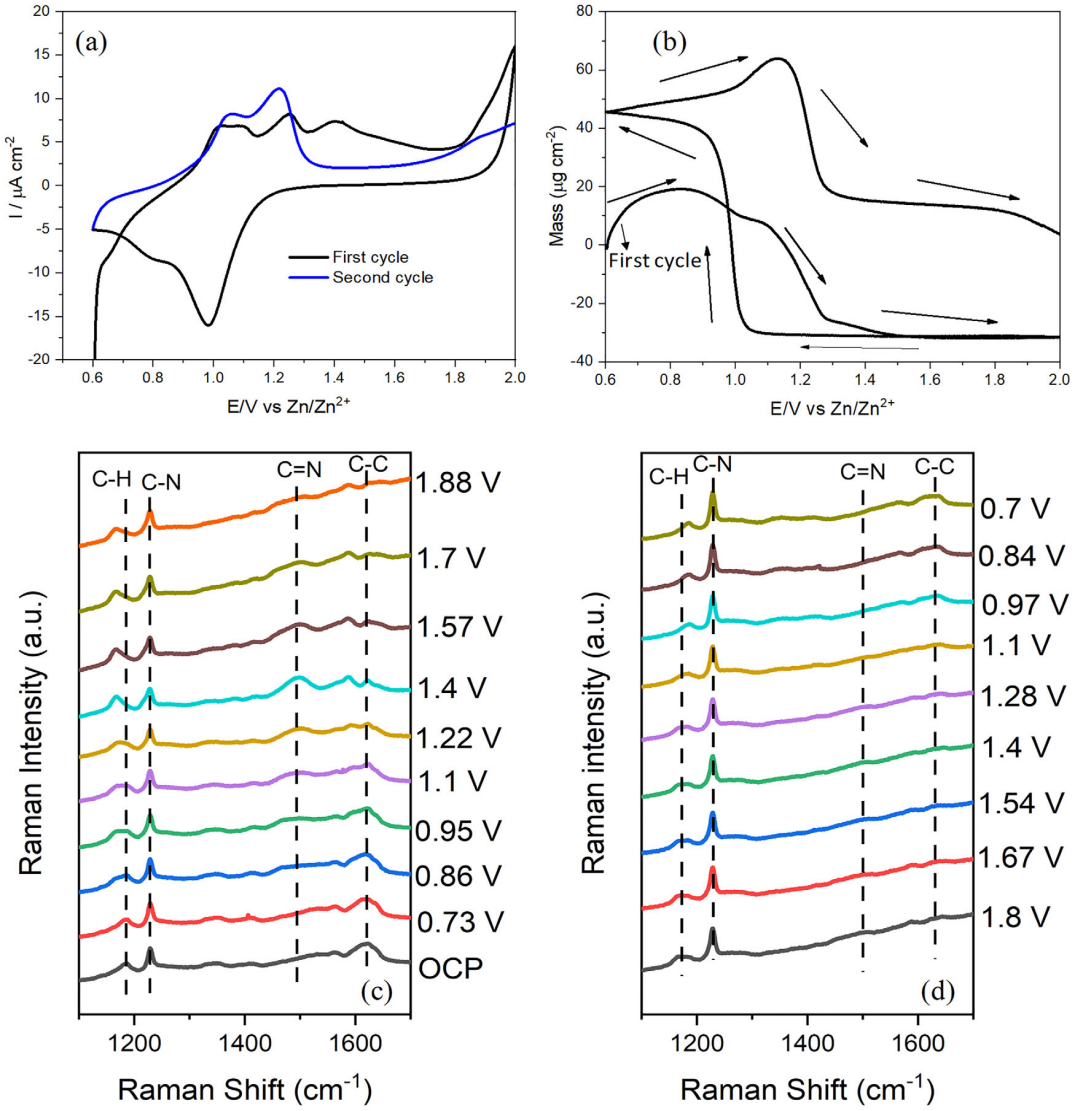
a) Cyclic voltammogram and b) corresponding mass change of Zn interaction with doped‐PANI during cyclic voltammetry in the potential range of 0.6–2.0 V in 2 m Zn(TfO)_2_ electrolyte; (c,d) Corresponding in situ Raman spectra of doped‐PANI during CV.

The EQCM‐Raman experiments were also performed for PANI/TfO_25_ film. **Figure** **4**a,b presents the cyclic voltammogram and the corresponding mass change, respectively. In contrast to the CV of PANI, PANI/TfO_25_ film displayed a more complex redox process with oxidation peaks ≈1.0, 1.09, 1.25, and 1.4 V during the first scan. During the cathodic scan, two reduction peaks are observed at 1.0 and 0.8 V. This redox process indicates the transformation of PANI to its different oxidation states, accompanied by the insertion and extraction of Zn^2+^ ions. Corresponding EQCM measurements show a mass increase up to 1.2 V, which can be related to the insertion of triflate anions to maintain charge neutrality during anodic scan. After 1.2 V, a mass decrease is observed, likely due to ion expulsion or rearrangement during transition to the more oxidized state, along with deintercalation of Zn. On the cathodic scan, a sharp and noticeable mass increase ≈1.0 V strongly suggests Zn^2+^ intercalation into the PANI matrix (Figure 4b).^[^ ^35^ ^]^ The same behavior was observed in the second cycle, with a mass increase at ≈1.0 V attributed to anion insertion, followed by a mass decrease corresponding to Zn^2+^ extraction. The higher current response observed in the CV of PANI/TfO_25_, along with the greater mass changes recorded by EQCM compared to those of the PANI (Figure 3a,b), indicate that the Zn^2+^ intercalation/ deintercalation process occurs more readily in the doped‐PANI film. Figure 4c,d shows in situ Raman spectra recorded at different electrode potentials during anodic and cathodic scans, respectively. At open circuit potential (OCP), characteristic PANI bands are observed at 1185 cm^−1^ (C─H bending of the benzenoid ring), 1228 cm^−1^ (benzene‐ring deformation vibration), 1564 cm^−1^ (C─C stretching in benzenoid ring), and 1622 cm^−1^ (C─C stretching vibrations of the benzenoid ring), indicating the predominance of the emeraldine salt form.^[^ ^32^, ^33^ ^]^ Initially, there is no peak at 1493 cm^−1^, which corresponds to C═N stretching vibrations in quinonoid units. Upon increasing the potential from 0.95 to 1.7 V, a distinct band emerges at 1494 cm^−1^, which is typically assigned to the quinoid ring structure and reflects the transition from emeraldine to the more oxidized pernigraniline form. Concurrently, the C═C stretching band shifts from 1564 to 1587 cm^−1^, suggesting enhanced quinoid character and increased conjugation length within the polymer backbone. Notably, the band at 1228 cm^−1^ exhibits progressive broadening, while the C─H bending vibration at 1185 cm^−1^ shifts to the lower wavenumbers as the potential increases. These spectral changes are likely associated with modifications in bond conjugation and alteration in the electron density distribution along the polymer film, which can be attributed to the interaction/deintercalation of Zn^2+^ ions.

Unlike pristine PANI, which retained its oxidized structure during the cathodic scan, the PANI/TfO_25_ film demonstrated a reversible structural change, with the quinoid‐related bands diminishing and the benzenoid features re‐emerging upon reduction. The C═N stretching band at 1494 cm^−1^, which emerged during the anodic scan, disappeared as the potential decreased. The C═C stretching band shifted to lower wavenumbers upon oxidation and returned to its original position, indicating the reversion of benzenoid and quinoid structures characteristic of the emeraldine and leucoemeraldine forms (Figure 4d). These comparative results highlight the role of TfO^−^ in tuning both the ion‐exchange dynamics and the redox reversibility of PANI.

### 2.4 Cyclic Voltammetry Studies

**Figure 5:**
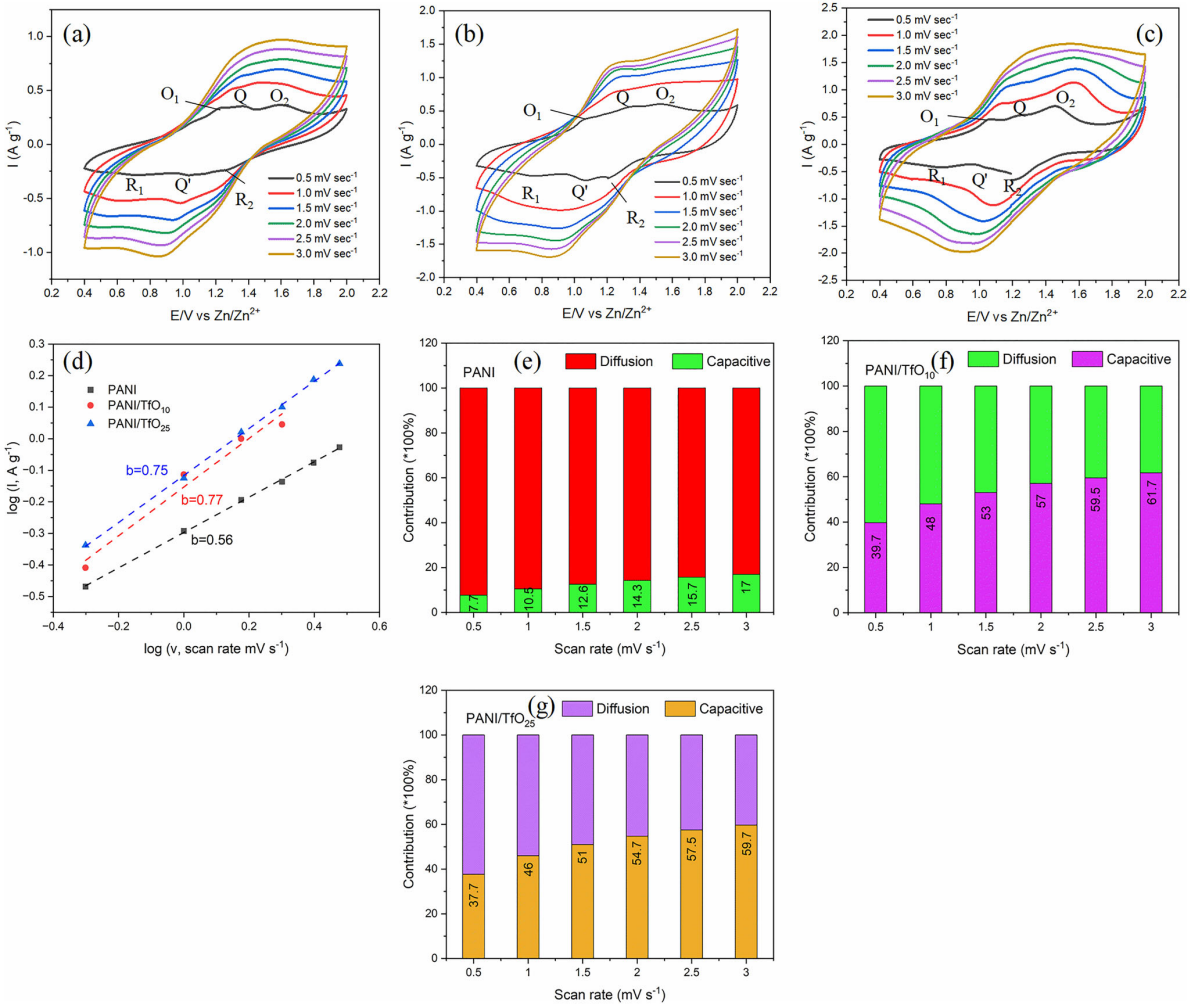
CV curves of a) PANI, b) PANI/ TfO_10_, c) PANI/ TfO_25_ in 2 M Zn(TfO)_2_ aqueous electrolyte at different scan rates; d) relationship between log i and log υ plots of CVs; diagram of the capacitive contribution to the total capacity at different scan rates of e) PANI, f) PANI/TfO _10_, g) PANI/TfO_25_.

The CVs of PANI and doped‐PANI in 2 m Zn(TfO)_2_ electrolyte at various scan rates are presented in **Figure** **5**a–c. The CVs are similar to those observed on Au in the EQCM studies. At low scan rate, the CVs of all electrodes exhibit two pairs of redox peaks (labeled O_1_‐R_1_ and O_2_‐R_2_), along with a smaller middle peak denoted as Q'–Q. The redox peaks at ≈1 and 1.4 V can be attributed to the leucoemeraldine‐emeraldine and emeraldine‐pernigraniline redox reactions of PANI, respectively, together with Zn intercalation/deintercalation. The middle peak is associated with reactions of quinone sites within the polymer structure.^[^ ^12^, ^36^ ^]^ To evaluate the contribution of capacitive and diffusion‐controlled processes, a plot of log current vs log scan rate was used based on Equations (1) and (2)

(1) $$i=av^{b}$$

(2) logI=loga+blogv

where a is a constant and b is the slope indicative of the charge storage mechanism. The b value of 0.5 typically indicates a diffusion‐controlled process, commonly observed in battery‐type behavior, whereas the b value approaching 1 suggests a surface‐controlled capacitive process. From Figure 5d, it can be seen that the value of b was found to be 0.56, 0.77, and 0.75 for PANI, PANI/TfO_10_, and PANI/ TfO_25_, respectively, suggesting that the anion doping enhances the capacitive contribution of the charge storage process. The capacitive and faradaic contribution for different PANI is shown in Figure 5e–g. The incorporation of an anion into the PANI matrix resulted in a notable enhancement of its capacitive behavior. In the PANI/TfO_25_, the capacitive contribution increased from 37.7% at 0.5 mV s^−1^ to 59.7% at 3 mV s^−1^ (Figure 5g). This increase in capacitive contribution with increasing scan rate is consistent with previous observations.^[^ ^12^, ^37^ ^]^

**Figure 6:**
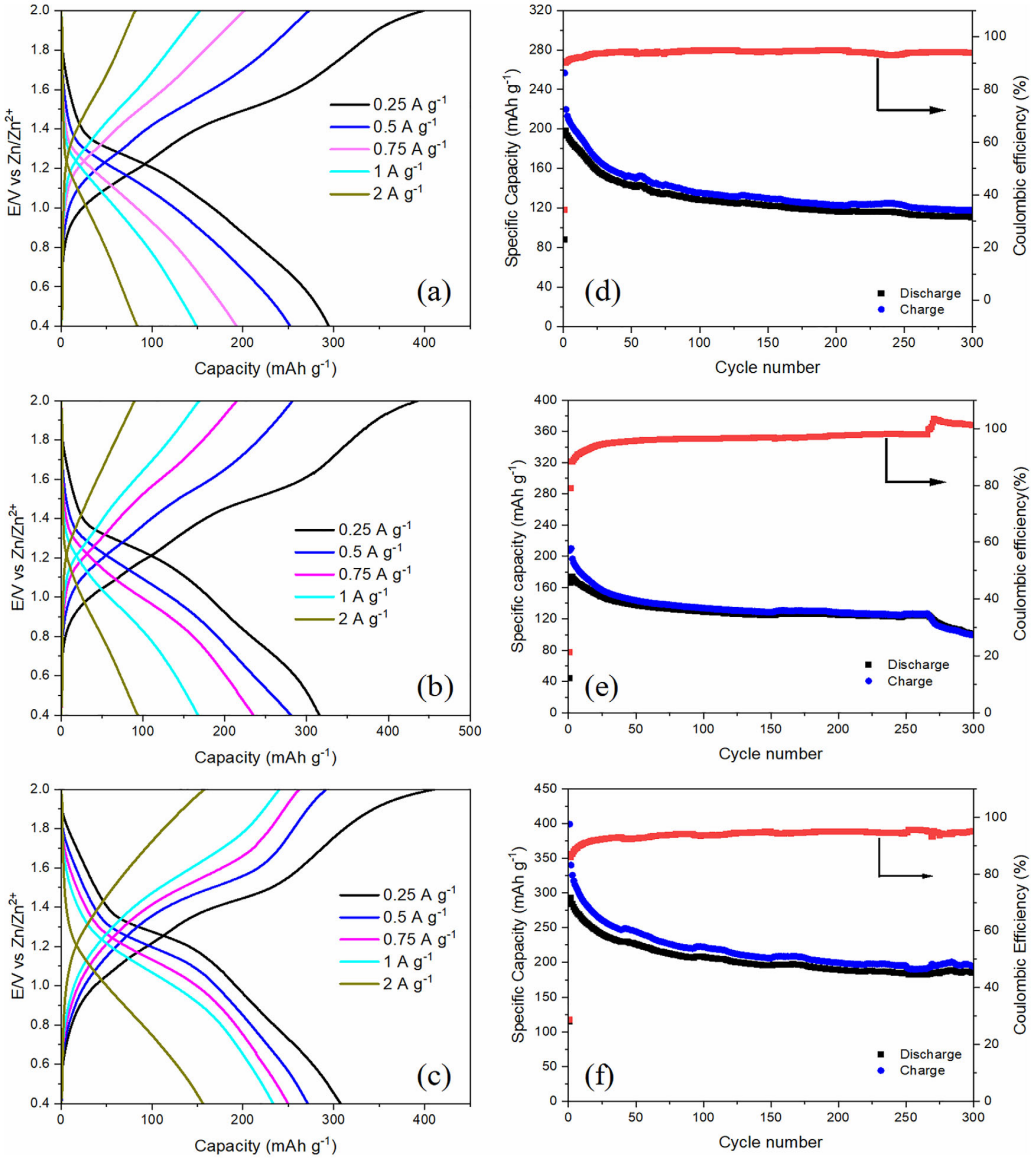
Galvanostatic charge/discharge curves and capacity and coulombic efficiency evolution at 1 A g^−1^ of the PANI (a,d), PANI/TfO_10_ (b,e), and PANI/TfO_25_ (c,f) cathodes in 2 M Zn(TfO)_2_.

To investigate the effect of anion‐doping on the capacity of PANI film, charge/discharge studies were performed. **Figure** **6**a–c illustrates the galvanostatic charge/discharge profiles of PANI, PANI/TfO_10_, and PANI/TfO_25_ electrodes, respectively, measured at various current densities ranging from 0.25 to 2 A g^−1^ in a 2 m Zn(TfO)_2_ aqueous electrolyte. The undoped PANI electrode exhibits a specific capacity of 150 mAh g^−1^ at a current density of 1 A g^−1^, whereas the PANI/TfO_10_ and PANI/TfO_25_ electrodes deliver enhanced capacities of 165 and 235 mAh g^−1^, respectively, with the latter representing the highest value reported to date for PANI‐based electrodes in zinc‐ion batteries.^[^ ^12^, ^22^, ^36^, ^38^, ^39^, ^40^, ^41^ ^]^ With the increase in current density from 0.25 to 2.0 A g^−1^, the specific capacities of all batteries gradually decrease. It is noteworthy that the PANI/TfO_25_ electrode shows significantly less capacity loss compared to the other electrodes, maintaining a high specific capacity of 100 mAh g^−1^ even at a current density of 2 A g^−1^. Below 10 mM doping concentration, the capacity is lower than that of pristine PANI (Figure S5, Supporting Information). The low doping concentration might have partially blocked the active sites or disrupted the charge transport within the polymer matrix. Increasing the doping concentration to 50 mM resulted in a decline in the performance, which may be caused by the decreased conductivity in the PANI film (Figure S6, Supporting Information).

The long‐term cyclic behaviors of PANI, PANI/TfO_10,_ and PANI/TfO_25_ electrodes were performed at 1 A g^−1^ (Figure 6d–f). The PANI/TfO_25_ electrode demonstrates remarkable cycling stability, retaining a capacity of >175 mAh g^−1^ with 85% capacity retention after 300 cycles (Figure 6f), which is superior to the PANI and PANI/TfO_10_ electrodes with only 120 mAh g^−1^ and 100 mAh g^−1^ capacity left after 300 cycles (Figure 6d,e). The Coulombic efficiency for all electrodes remained slightly below 100% during extended cycling, which may be attributed to minor parasitic reactions in the aqueous electrolyte and partial irreversibility of Zn^2+^ ion interaction with the PANI film. Moreover, a sudden drop in the capacity of the PANI/TfO_10_ electrode was observed after 270 cycles (Figure 6e). This may be attributed to the gradual degradation of the PANI structure, including overoxidation or mechanical fatigue, which could lead to a loss of conductivity or a reduction in the electrochemically active regions.^[^ ^42^ ^]^

**Figure 7:**
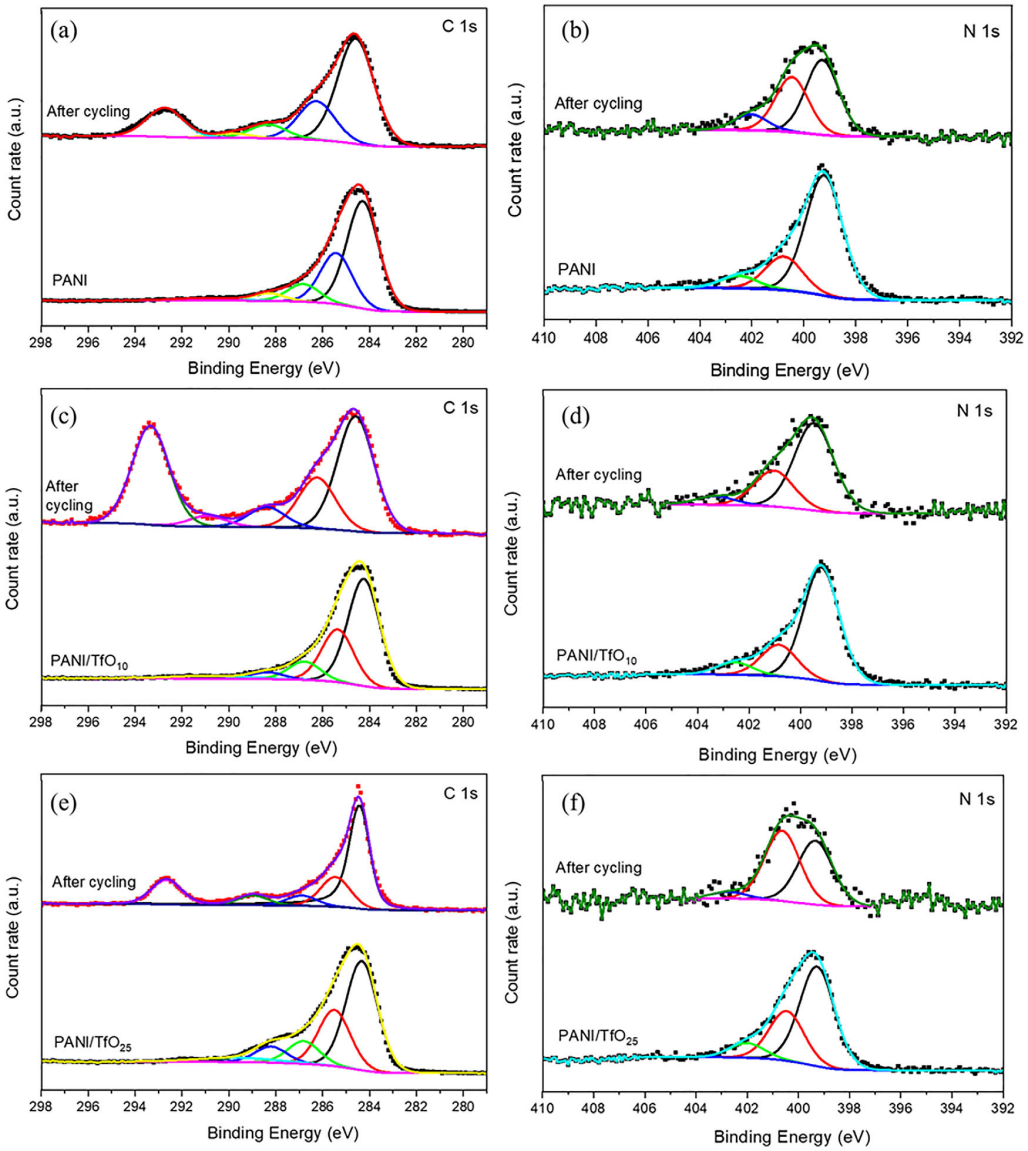
Detailed C 1s, and N 1s spectra of the as‐prepared and after a–b) cycling PANI, c–d) PANI/TfO_10_, and e,f) PANI/TfO_25_ films.

To further understand the changes in the PANI and doped‐PANI structure, ex situ XPS was performed. **Figure** **7** shows the detailed XPS spectra of C 1s and N 1s, reflecting the changes in the polymer films after the cycling. In all films, C 1s spectra typically exhibit peaks at 284.3 eV corresponding to the binding energy of the aromatic C─C and C═C, with additional components ≈285.4, 286.8, and 288.3 eV attributed to the C─N, C═O (C─N^+^), and COO binding energy (Figure 7a,c,e).^[^ ^13^, ^29^ ^]^ After the cycling process, the peaks at 292.8, 293.3, and 292.7 eV observed for PANI, PANI/TfO_10,_ and PANI/TfO_25_ attributed to C–F increased significantly, suggesting more TfO^−^ anion insertion into the polymer matrix (Figure 7a,c,e ). All binding energy peaks shifted toward higher binding energy, and two new peaks attributed to Zn 2p were observed (Figures S7–S9, Supporting Information). In the N 1s XPS spectrum of the samples, three main peaks were observed at 399.2, 400.7, and 402.5 eV corresponding to quinoid imine (─N═), benzenoid amine (─NH═), and the positively charged polaron species (─NH^+^═), respectively (Figure 7b,d,f).^[^ ^26^ ^]^ For doped‐PANI films, the latter two peaks shift to higher binding energies, consistent with in situ Raman results and suggesting an increased oxidation level and polaron/bipolaron formation.

Based on the above analysis, the incorporation of Zn(TfO)_2_ during the electropolymerization of PANI not only modified the morphological and electronic structure of the polymer but also significantly enhanced its electrochemical stability and capacity retention during cycling. The structural insights obtained through ex situ XPS analysis confirm that such improvements are closely related to the coordinated interaction between TfO^−^ anions and the PANI backbone, as well as the redistribution of oxygen species within the film.

**Figure 8:**
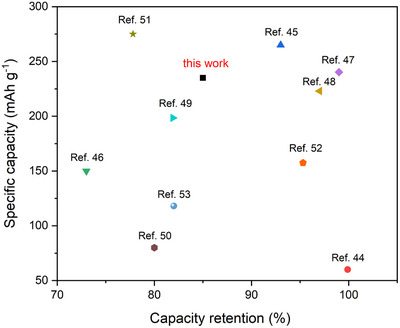
Comparison of the electrochemical performances of PANI/TfO_25_ with recently reported cathode materials.

To further highlight the electrochemical performance of the PANI/TfO_25_ electrode, the specific capacity and capacity retention were compared with recently reported cathode materials (metal oxides, polymers) in **Figure** **8**.^[^ ^43^, ^44^, ^45^, ^46^, ^47^, ^48^, ^49^, ^50^, ^51^, ^52^ ^]^ It clearly demonstrates the competitive features of our results, supporting its potential application in aqueous ZIBs.

## 3 Conclusion

In conclusion, a series of PANI/TfO_x_ electrodes was successfully synthesized via electropolymerization onto carbon paper. A combined in situ EQCM‐Raman setup was employed for the first time to provide an in‐depth understanding of Zn intercalation and deintercalation behavior in PANI film. This novel approach allowed simultaneous monitoring of mass changes and molecular structural evolution on the same electrode, offering unique insights into the electrochemical processes. The results have shown that the PANI/TfO_25_ electrode exhibits more pronounced redox transitions and reversible mass variations, which are attributed to the weak coordinating nature of the TfO^−^ anions that facilitate ion transport. Additionally, the results were further validated through ex situ XPS analysis, establishing a comprehensive understanding. Our results demonstrate that the incorporation of TfO^−^ ions enhances both the capacity and cycling stability of PANI. Notably, the PANI/TfO_25_ cathode delivers a high specific capacity of 235 mAh g^−1^ at a current density of 1 A g^−1^, surpassing that of pure PANI. In addition, it exhibits excellent rate capability and long‐term stability, maintaining 85% of its initial capacity after 300 cycles. Nevertheless, when the dopant concentration exceeded an optimal threshold, both the capacity and the cycling performance declined, likely due to excessive ionic interference or structural distortion. These results suggest that a balanced anion content is crucial for achieving optimal electrochemical performance in PANI cathodes. These results may encourage further modification of conducting polymers for high‐performance ZIBs.

## 4 Experimental Section

Aniline monomer (Thermo Scientific, 99.8%), sulfuric acid (Fisher Chemical, 98%), and zinc triflate [Zn(TfO)_2_] (Thermo Scientific, 98%) were used as received.

PANI and doped‐PANI were synthesized using electrodeposition. Cyclic voltammetry (CV) was performed in the potential ranges of −0.5–1.2 V at a scan rate of 10 mV s^−1^ in a conventional single‐compartment electrochemical cell employing a three‐electrode configuration where Ag/AgCl, carbon paper (CP), and carbon plate served as reference, working, and counter electrodes, respectively. Electrodeposition was performed in a mixture of 0.1 m H_2_SO_4_ and 0.1 m aniline with and without the addition of Zn(TfO)_2_ with varying concentrations (5, 10, 25, and 50 mm). After deposition, films were rinsed with D.I water and dried in a vacuum oven at 60 °C for 2 h. Furthermore, mass change during anodic and cathodic processes was investigated using EQCM.

Electrochemical measurements were performed in a home‐built cell containing PANI/doped‐PANI as the cathode, 2 m Zn(TfO)_2_ as electrolyte, selected due to its wide electrochemical stability window and good compatibility with PANI, and a Zn foil as anode, respectively. Before the experiments, the Zn foils were mechanically polished to remove the oxide layer. Cyclic voltammetry (CV) and galvanostatic charge–discharge (GCD) experiments were carried out using a Biologic VSP‐3e potentiostat/galvanostat, and long‐term cycling experiments were performed using a battery tester (NanoCycler). EQCM measurements were performed with a Gamry Instruments eQCM 15 m having a resolution of 0.02 Hz with a Gamry Instrument Interface 1010 B potentiostat/galvanostat. A 14 mm diameter 5 MHz AT‐cut quartz crystal with one side coated with Cr/Au was used as the working electrode (QuartzPro, Sweden AB). The sensitivity factor was evaluated by electrodeposition of Zn. To calculate the mass change by EQCM, electrochemical deposition of PANI and doped‐PANI was performed on an Au quartz crystal with a Pt wire auxiliary electrode and Ag/AgCl reference electrode. Given the thin PANI film, the resonance frequency shift (∆f) was converted into mass change (∆m) on the working electrode surface using the Sauerbrey equation, assuming negligible viscoelastic effects.^[^ ^53^ ^]^

(3) $$\Delta m\left(g\right)=-1.6\times 10^{-8}\cdot \Delta f\left(\mathrm{Hz}\right)$$

For in situ EQCM‐Raman analysis, a bespoke cell from Redox.me was used. The cell was assembled under a Renishaw inVia confocal Raman microscope. The Raman microscope was equipped with a 514 nm laser (Stellar‐REN) and used a diffraction grating of 1800 lines mm^−1^ with a Renishaw CCD camera as the detector, respectively. The Raman spectroscopy and EQCM measurements were acquired simultaneously while running the CV.

The surface morphology of PANI was examined by scanning electron microscope (SEM) (Jeol IT200LV, USA), which is coupled with an energy dispersive spectroscopy (EDS) (Oxford EDS) as the detector.

XPS Analysis was performed using a Thermo NEXSA XPS fitted with a monochromated Al kα X‐ray source (1486.7 eV), a spherical sector analyzer, and 3 multichannel resistive plates, 128 channel delay line detectors. All data was recorded at 19.2 W and an X‐ray beam size of 400 × 200 µm. Survey scans were recorded at a pass energy of 200 eV, and high‐resolution scans were recorded at a pass energy of 40 eV. Electronic charge neutralization was achieved using a Dual‐beam low‐energy electron/ion source (Thermo Scientific FG‐03). Ion gun current = 150 µA. Ion gun voltage = 45 V. All sample data were recorded at a pressure below 10^−8^ Torr and a room temperature of 294 K. Data were analyzed using CasaXPS v2.3.20PR1.0.

## Conflict of Interest

The authors declare no conflict of interest.

## Supporting information

Supporting Information

## Data Availability

The data that support the findings of this study are openly available in Brunel figshare at 10.17633/rd.brunel.29383454, reference number 29383454.
