# Supplementary material for: Combined In Situ EQCM‐Raman Study of Zn Storage Mechanism in Polyaniline for Zinc‐Ion Battery
Source: Small Methods. 2025 Oct 1;9(11):e01273. doi: 10.1002/smtd.202501273 (PMC12641370; doi:10.1002/smtd.202501273)
Supplement: Supplementary file 1 — Supporting Information [file SMTD-9-e01273-s001.docx]

**Supporting Information**

**Combined In Situ EQCM-Raman Study of Zn Storage Mechanism in Polyaniline for Zinc-Ion Battery**

Emine Kapancik Ulker^1,2^, Pranay Hirani^1^, Shaoliang Guan^3^, Abhishek Lahiri^1^,

^1^Department of Chemical Engineering, Brunel University London, Uxbridge, UB8 3PH, UK

^2^Department of Chemistry, Faculty of Science and Arts, Recep Tayyip Erdogan University, Rize, 53100, Turkey

^3^Maxwell centre, Cavendish Laboratory, J J Thomson avenue, Cambridge, CB3 0HE

**
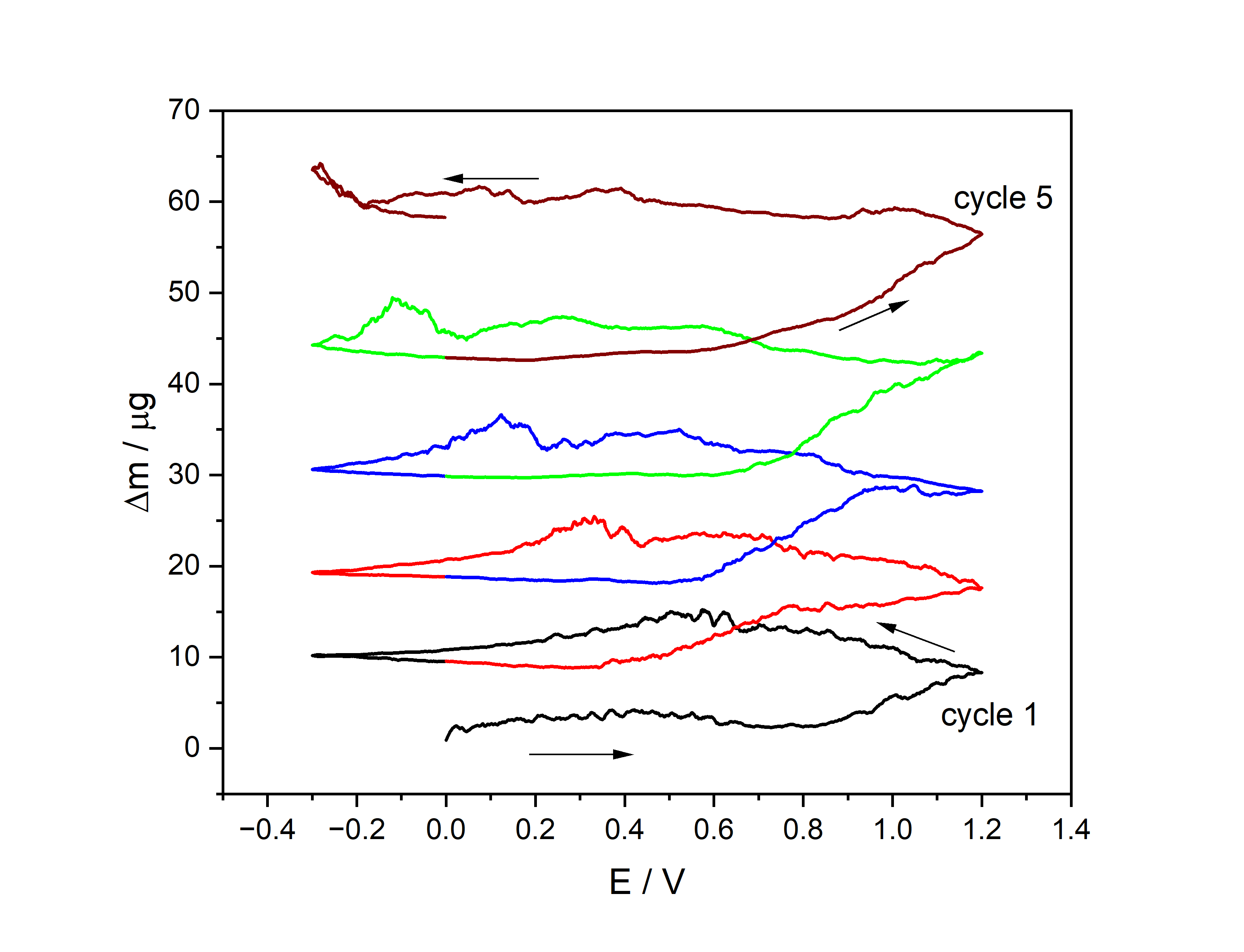
**

**Figure S1.** Δm vs. E from EQCM data for the deposition of a PANI film.

**
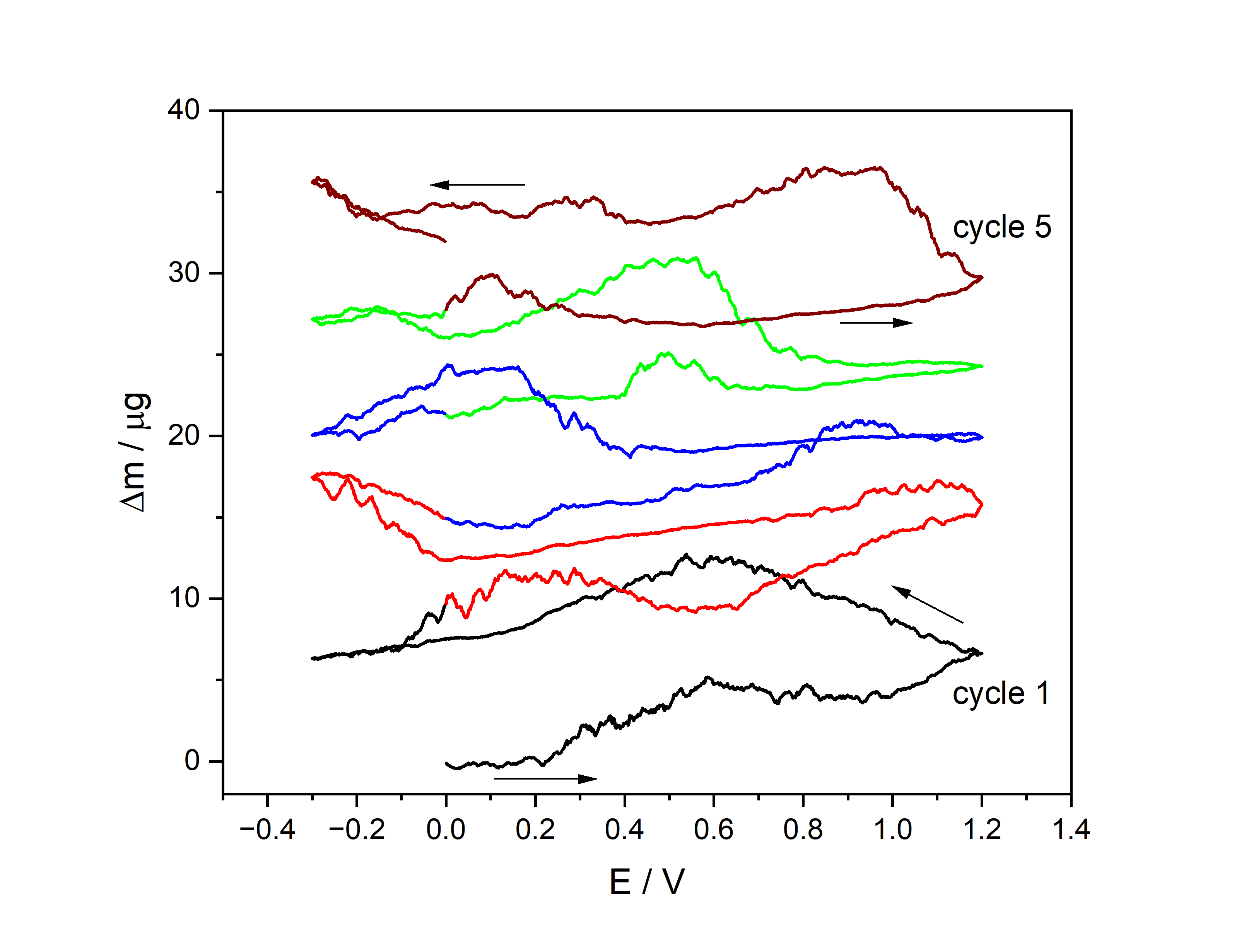
**

**Figure S2.** Δm vs. E from EQCM data for the deposition of a PANI/TfO_25_ film.


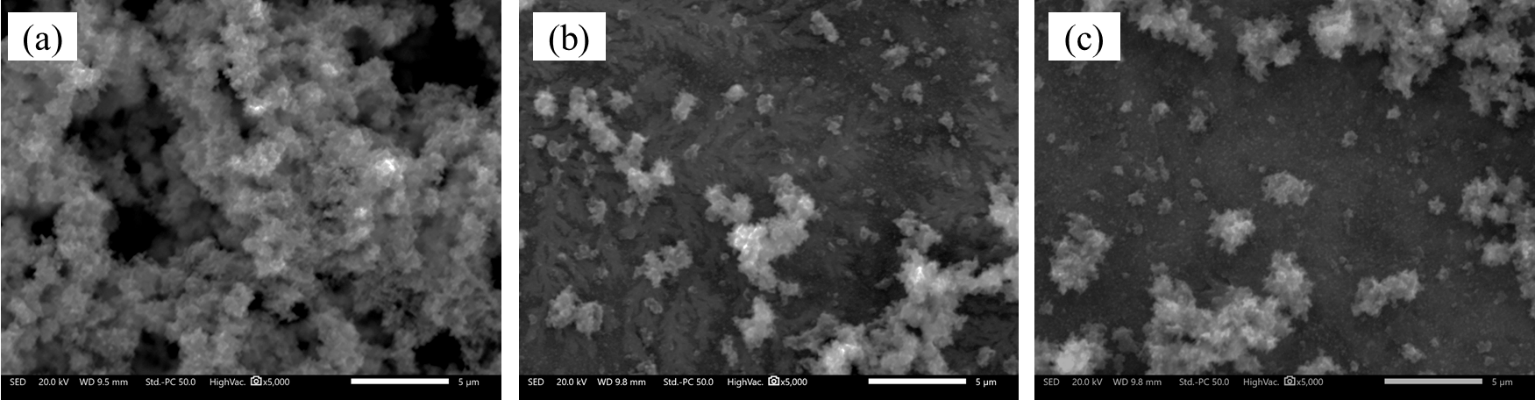


**Figure S3.** SEM images of a) PANI, b) PANI/TfO_10_ and c) PANI/TfO_25_

**
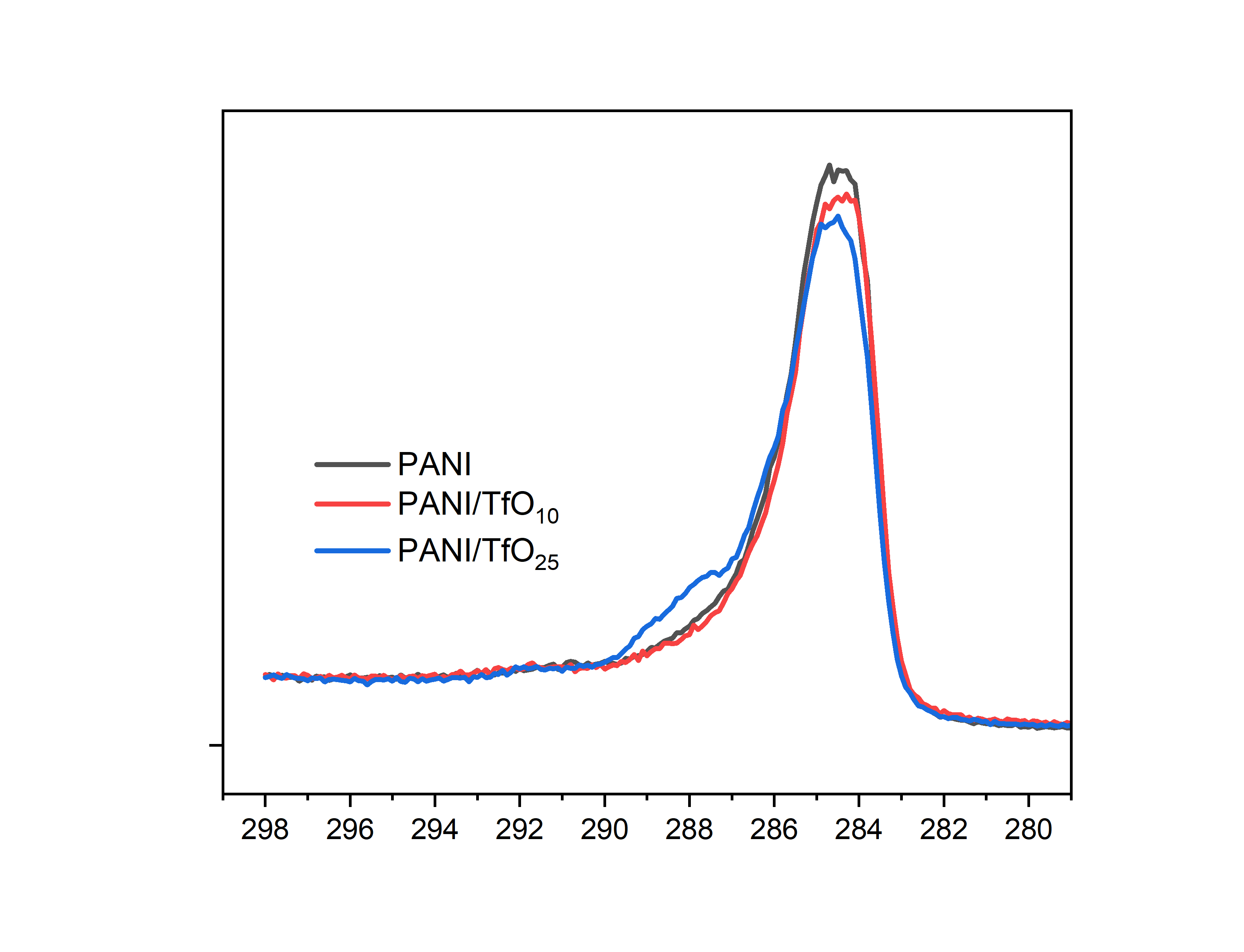
**

**Figure S4.** Comparison of C 1s XPS spectra of the PANI and doped-PANI films.

**Figure S5.** Galvanostatic charge/ discharge curves of the PANI/TfO_5_ electrode

**Figure S6.** Galvanostatic charge/ discharge curves of the PANI/TfO_50_ electrode


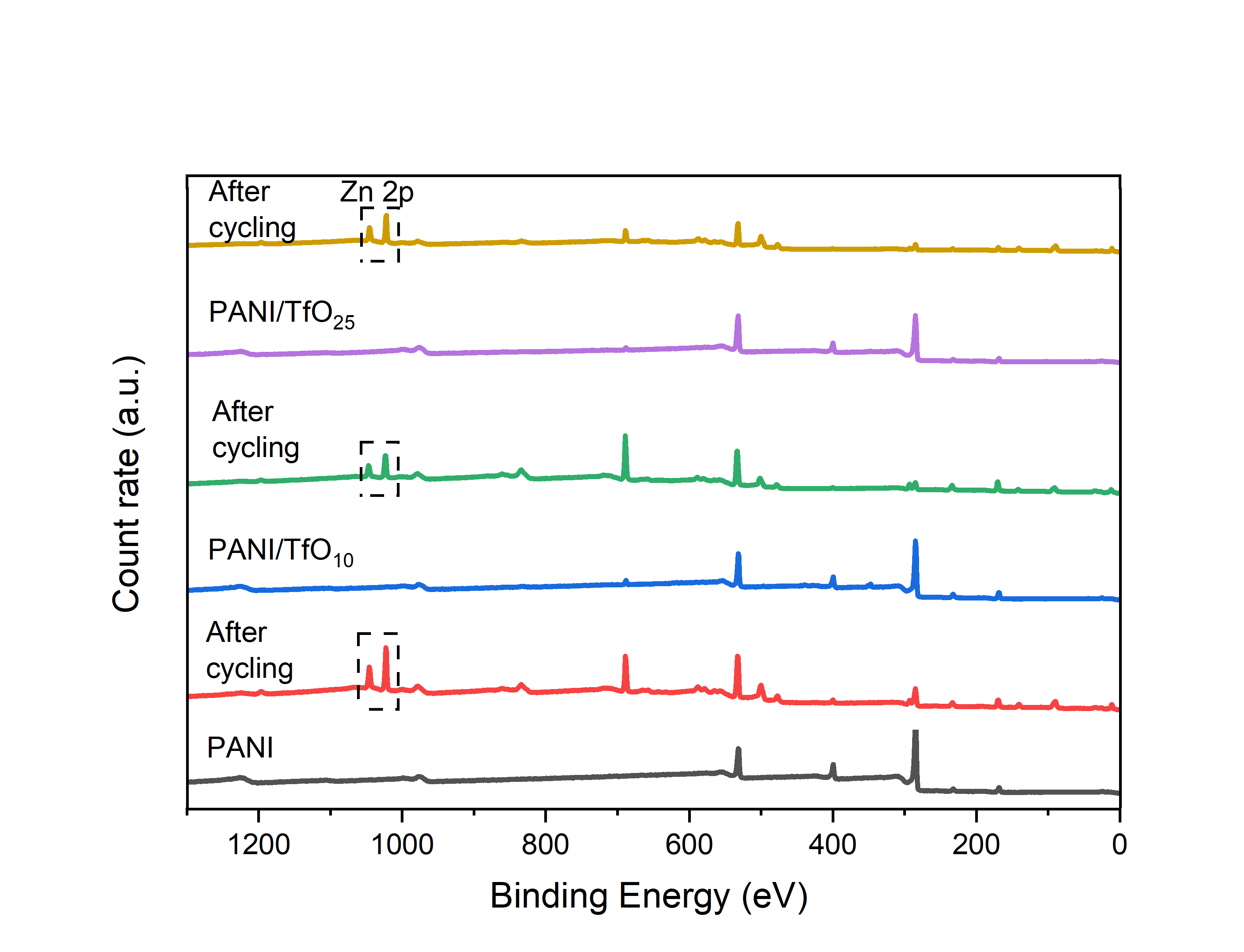


**Figure S7.** Comparison of the survey XPS spectra of as-prepared and after cycling PANI, PANI/TfO_10_ and PANI/TfO_25_ films.


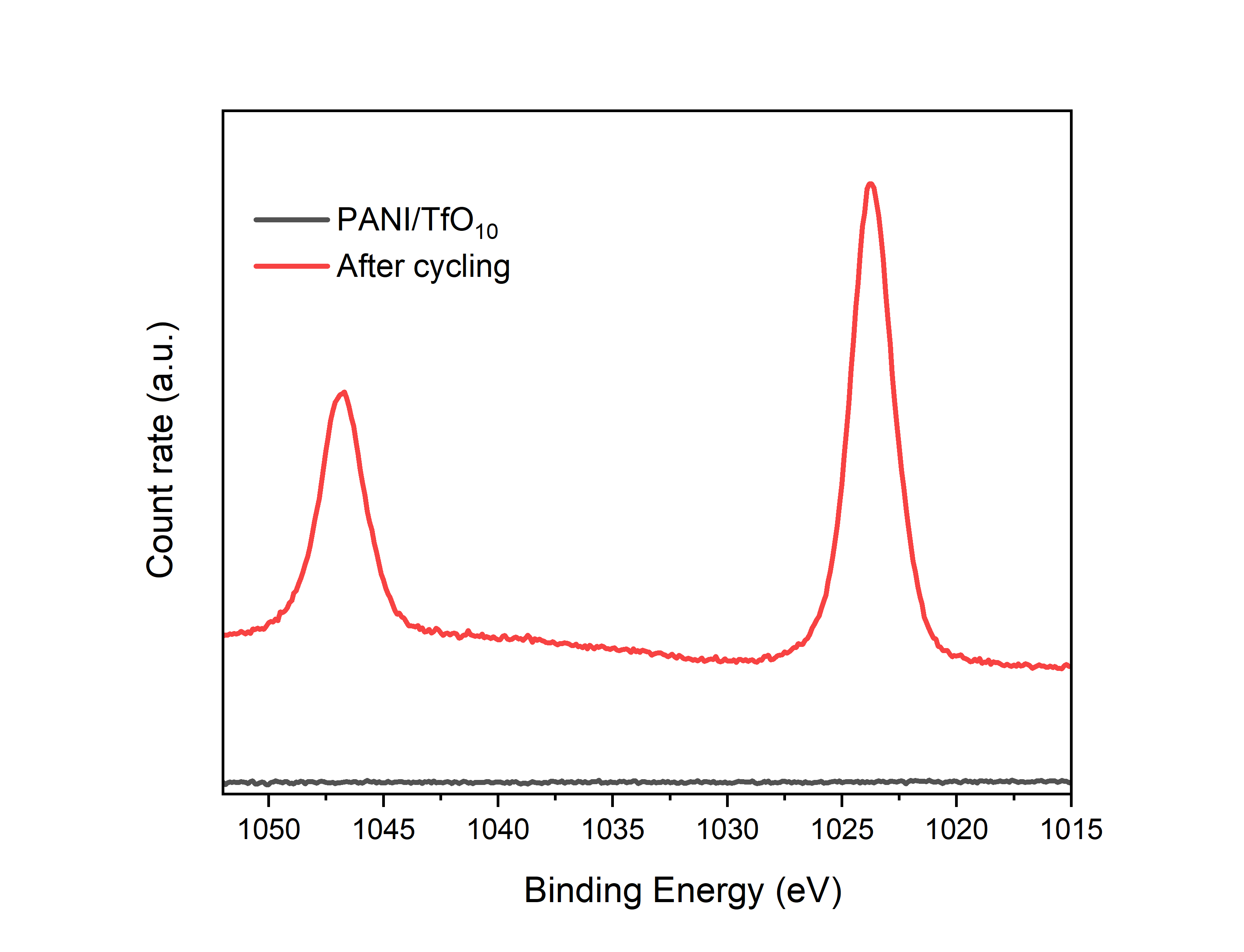


**Figure S8.** Detailed XPS spectra of Zn 2p for PANI/TfO_10_ after 300 cycles in 2M Zn(TfO)_2_ electrolyte


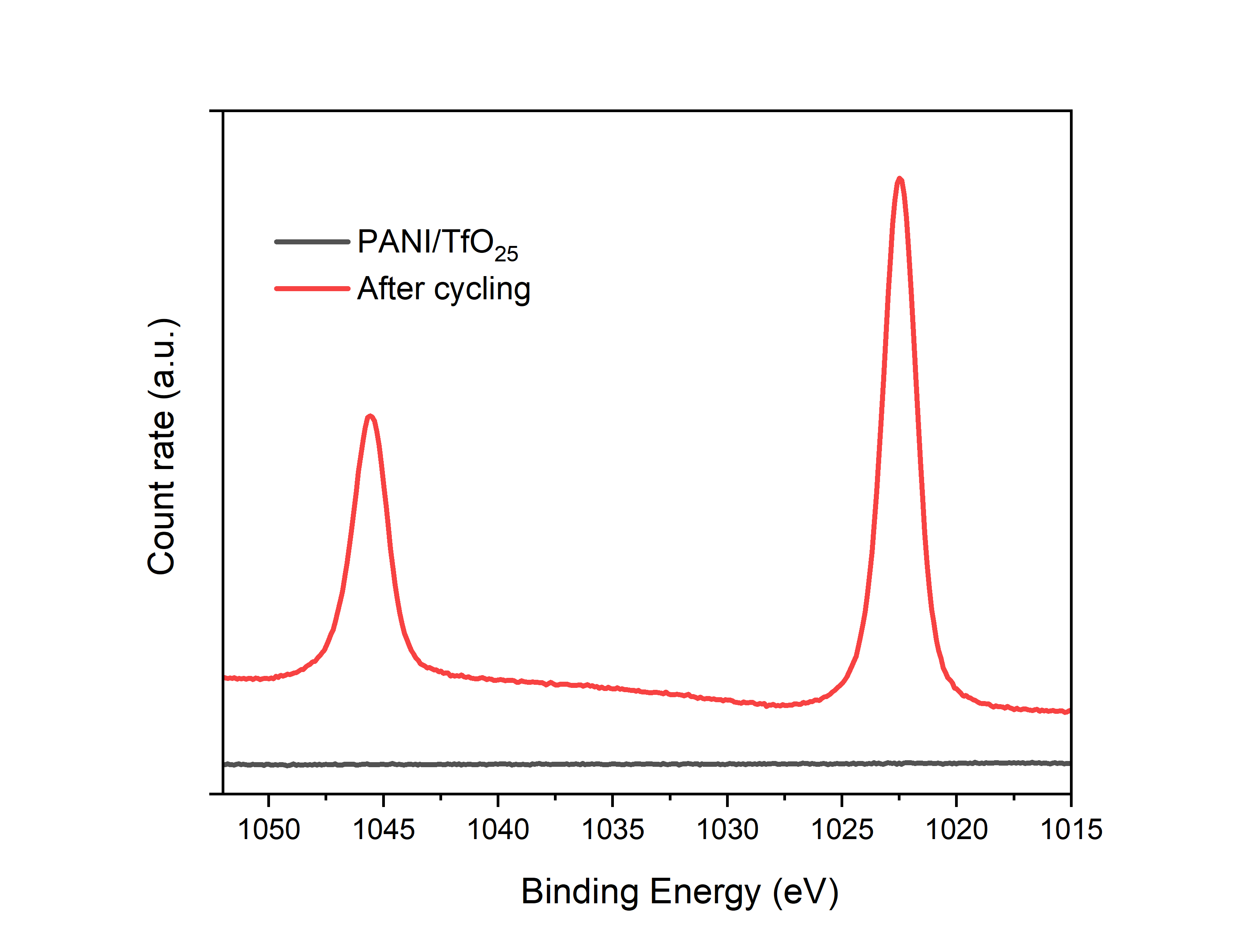


**Figure S9.** Detailed XPS spectra of Zn 2p for PANI/TfO_25_ after 300 cycles in 2M Zn(TfO)_2_ electrolyte
